# Latent JAK2 V617F-Positive Myeloproliferative Neoplasm With Normal Blood Counts and Recurrent Splanchnic Vein Thrombosis in a Young Woman

**DOI:** 10.7759/cureus.85208

**Published:** 2025-06-01

**Authors:** Aye M Thida, Carol Luhrs, Aastha Baral, Li Zhonghua, Jordonna Brown

**Affiliations:** 1 Hematology and Medical Oncology, SUNY Downstate Health Sciences University, Brooklyn, USA; 2 Pathology, SUNY Downstate Health Sciences University, Brooklyn, USA; 3 Pathology, Kings County Hospital Center, New York City, USA

**Keywords:** jak2 v617f-positive, latent jak2 v617f-positive myeloproliferative neoplasm, latent mpn, myeloproliferative neoplasm disease, splanchnic vein thrombosis

## Abstract

Latent myeloproliferative neoplasms are diagnostically challenging clonal hematopoietic disorders characterized by the JAK2 V617F mutation without overt hematologic abnormalities. This case report describes a 33-year-old woman presenting with recurrent splanchnic vein thrombosis, splenomegaly, and a history of unprovoked pulmonary embolism, found to have a JAK2 V617F-positive latent myeloproliferative neoplasm. Despite normal blood counts and unremarkable bone marrow findings, molecular testing confirmed the JAK2 V617F mutation (17% allele frequency), highlighting its critical role in diagnosing occult myeloproliferative neoplasms in patients with unexplained thrombosis. The patient’s recurrent thrombotic events, including portal and mesenteric vein thrombosis, underscore the prothrombotic phenotype driven by JAK2 V617F mutation. Management included indefinite anticoagulation with apixaban, low-dose aspirin, and hydroxyurea to mitigate thrombotic risk and address the underlying clonal process. This case emphasizes the importance of molecular testing for JAK2 V617F in young patients with recurrent or unusual-site thrombosis, even with normal hematologic parameters, and the need for tailored therapeutic strategies to prevent complications and monitor disease progression.

## Introduction

Myeloproliferative neoplasms (MPNs) are clonal hematopoietic stem cell disorders characterized by excessive proliferation of myeloid lineages - granulocytic, erythroid, or megakaryocytic - resulting in elevated peripheral blood counts [1]. Common Philadelphia chromosome-negative MPNs, including polycythemia vera, essential thrombocythemia, and primary myelofibrosis (MF), frequently harbor the JAK2 V617F mutation, detected in ~95% of polycythemia vera and 50-60% of essential thrombocythemia and primary MF cases [2-5]. This somatic mutation in exon 14 substitutes valine with phenylalanine at codon 617, causing constitutive JAK/STAT signaling and uncontrolled cell proliferation [2-5]. Latent or occult MPNs are a subset where patients carry the JAK2 V617F mutation but lack overt hematologic abnormalities, such as elevated blood counts, at presentation [6].

Latent MPNs often present with thrombotic events, particularly splanchnic vein thrombosis (SVT), which includes portal, hepatic, or mesenteric vein thrombosis [6]. SVT carries significant risks, such as portal hypertension, liver dysfunction, and recurrent thrombosis [7]. The JAK2 V617F mutation is identified in 32.7% of SVT cases and up to 49% of idiopathic SVT, emphasizing the need for molecular testing to uncover underlying MPNs in patients with normal blood counts [6]. These cases are diagnostically challenging due to the absence of typical MPN hematologic features, yet the thrombosis risk is significant, necessitating early diagnosis and tailored management [6]. This case report describes a 33-year-old woman with JAK2 V617F-positive latent MPN presenting with SVT, splenomegaly, and a history of unprovoked thrombosis, managed with anticoagulation, low-dose aspirin, and hydroxyurea. This report aims to elucidate the clinical presentation, diagnostic workup, and therapeutic strategies for JAK2 V617F-positive latent MPN, emphasizing the critical role of molecular testing and thrombosis prevention in such cases.

## Case presentation

A 33-year-old Black Hispanic woman presented to the emergency department with right upper quadrant (RUQ) pain for one day. Her medical history included anxiety disorder, an unprovoked pulmonary embolism five years prior, and portal vein thrombosis two years prior. She reported no family history of cancer, hematologic disorders, or thrombosis and denied smoking or alcohol use. Physical examination revealed RUQ tenderness and splenomegaly, with no other notable findings. Laboratory results showed a white blood cell (WBC) count of 3.81 K/µL (reference range: 4.50-10.90 K/µL), hemoglobin of 12.5 g/dL (reference range: 12.0-14.0 g/dL), and platelet count of 172 K/µL (reference range: 130-400 g/dL). The comprehensive metabolic profile, including liver and renal function, was unremarkable (Table 1).

**Table 1:** Summary of laboratory values

Parameters	Patient Values	Reference Range
Hemoglobin	12.5 g/dL	12-16 g/dL
White Blood Cell Count	3.81 K/uL	3.80-10.80 K/uL
Platelet Count	172 K/uL	150-400 K/uL
Alkaline Phosphatase	67 U/L	25-125 U/L
Alanine Transaminase	25 U/L	0-31 U/L
Aspartate Transaminase	20 U/L	10-35 U/L
Total Bilirubin	0.2 mg/dL	0.0-1.2 mg/dL
Creatinine	0.6 mg/dL	0.5-0.9 mg/dL
Estimated Glomerular Filtration Rate	>60 mL/minute/1.73 m^2^	>60 mL/minute/1.73 m^2^

**Figure 1:**
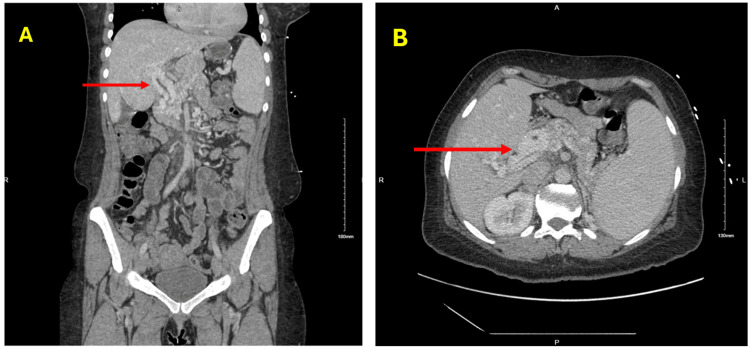
Computed tomography scan of the abdomen and pelvis with intravenous contrast, coronal (A) and axial (B) views demonstrating cavernous transformation of the portal vein (red arrows).

**Figure 2:**
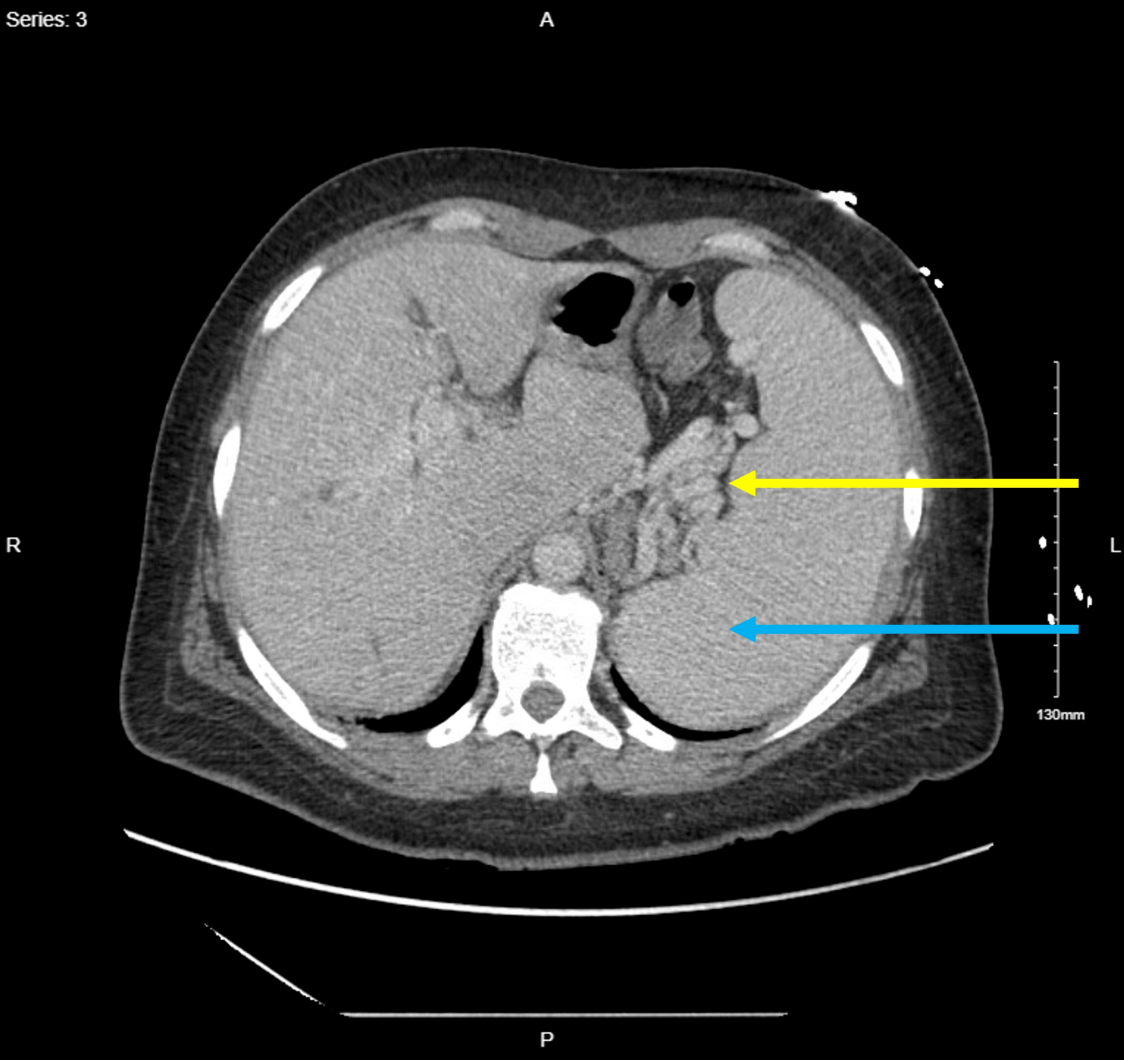
Computed tomography scan of the abdomen and pelvis with intravenous contrast, axial view demonstrating perisplenic veins (yellow arrow) and splenomegaly (blue arrow).

Computed tomography chest, abdomen, and pelvis with intravenous contrast ruled out pulmonary embolism but revealed redemonstration of cavernous transformation of the portal vein suggestive of chronic thrombosis, normal liver size and contour, perihepatic varices, splenomegaly (15 cm in craniocaudal dimension and 18 cm in anterior-posterior dimension), perisplenic, perigastric, and pericholecystic varices (Figures 1-2).

The patient disclosed inconsistent adherence to her prescribed apixaban therapy. Following an in-depth discussion of risks and benefits, she committed to ongoing treatment to prevent potentially life-threatening thrombotic events. During hospitalization, she received therapeutic enoxaparin, followed by a transition to apixaban 5 mg twice daily at discharge, with a plan for indefinite anticoagulation, subject to tolerability and regular follow-up.

**Figure 3:**
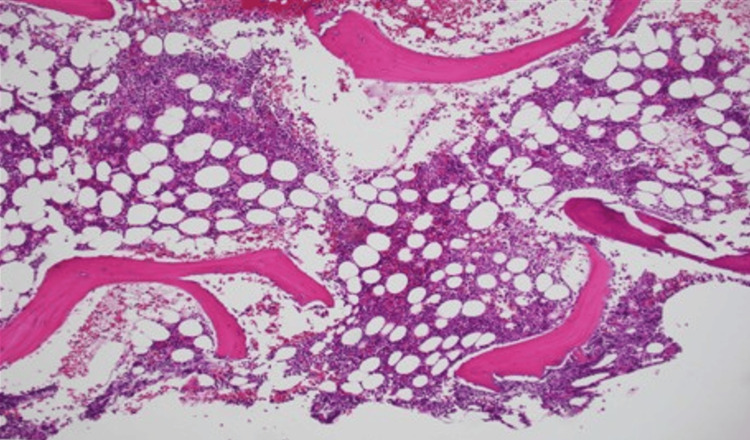
Bone marrow biopsy. Hematoxylin and eosin, original magnification 100×, showing normocellular marrow with trilineage hematopoiesis without any morphological dysplasia in the hematological cell lineages.

**Figure 4:**
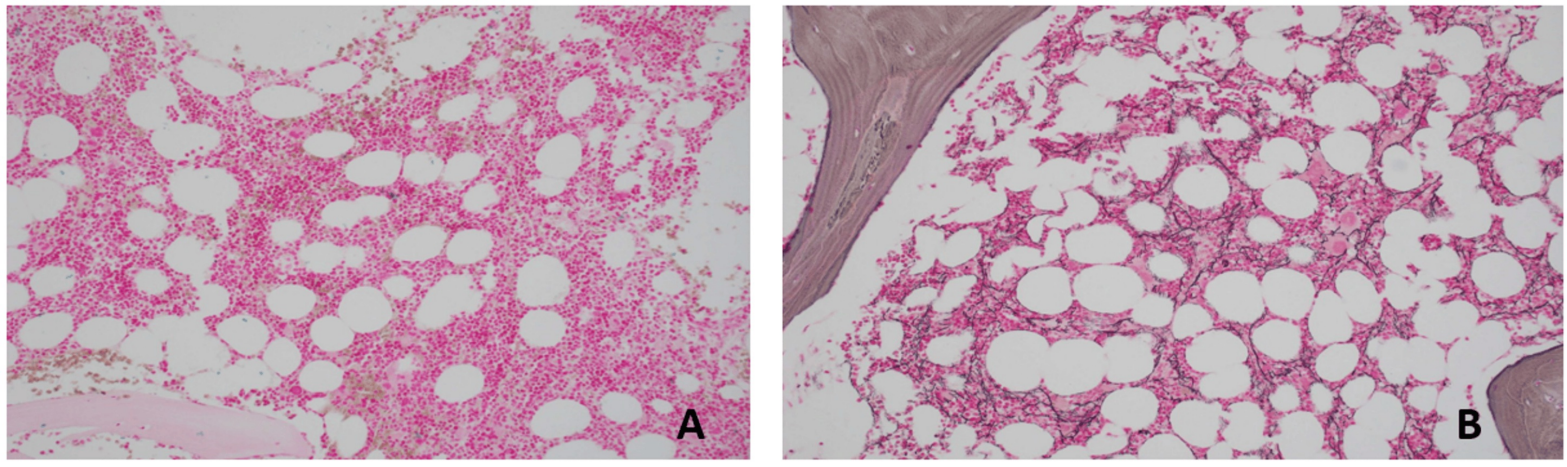
Bone marrow biopsy. (A) Iron stain demonstrating decreased stainable iron with no ring sideroblasts identified. (B) Reticulin stain showing a moderate increase in reticulin fibers with a loose distribution pattern.

At outpatient follow-up, a thrombophilia workup was initiated due to thrombosis in unusual sites. Tests for factor V Leiden, prothrombin gene mutations, protein C, protein S, antithrombin III, and antiphospholipid antibodies were negative. Peripheral blood testing for the JAK2 V617F mutation was positive. Complete blood count remained normal, and flow cytometry ruled out paroxysmal nocturnal hemoglobinuria. Bone marrow biopsy revealed normocellular marrow with trilineage hematopoiesis, full maturation, no increase in blasts, and no overt myeloid dysplasia (Figure 3). Iron staining indicated reduced stainable iron and no ring sideroblasts. Reticulin staining demonstrated moderate fibrosis, classified as grade MF-1 (reticulin 1+), characterized by a loosely distributed, diffuse meshwork of fibers without the dense intersections or coarse fiber bundles required for an MF-2 designation (Figure 4). Flow cytometry of the aspirate was normal, and cytogenetics revealed a normal female karyotype in 20 metaphases. Fluorescence in situ hybridization analysis, testing for BCR/ABL1, PML/RARA, RUNX1/RUNX1T1 [t(8;21)], CBFB [inv(16) or t(16;16)], and MLL (11q23) gene rearrangements detected no abnormalities. Next-generation sequencing (NGS) via the myeloid panel identified a Tier 1 JAK2 p.Val617Phe mutation (allele frequency: 17%), with no pathogenic gene fusions detected by the pan-heme fusion NGS panel. Serum erythropoietin was normal at 4.6 mIU/mL (reference range: 2.6-18.5).

Given the normal complete blood count, normal erythropoietin, and bone marrow findings, the patient did not meet the diagnostic criteria for polycythemia vera. Pre-fibrotic MF was excluded from the differential diagnosis due to normal megakaryocyte morphology, despite the presence of grade MF-1 fibrosis. The presence of splenomegaly posed a clinical challenge, as it could be attributed to either MPN or sequelae of splenic and portal vein thrombosis. The patient was started on aspirin 81 mg daily and continued on apixaban to reduce the risk of thrombosis recurrence. Following shared decision-making, hydroxyurea 500 mg every other day was initiated to address the underlying MPN and thrombotic risk.

At the three-month outpatient follow-up, the patient reported significant improvement in RUQ pain, with no recurrence of thrombotic events. Physical examination showed persistent splenomegaly, unchanged from baseline. The patient tolerated hydroxyurea 500 mg every other day, aspirin 81 mg daily, and apixaban 5 mg twice daily well, with no bleeding events or cytopenias.

## Discussion

This case illustrates a 33-year-old woman with JAK2 V617F-positive latent MPN presenting with recurrent SVT and normal blood counts, highlighting the diagnostic and therapeutic challenges of identifying occult MPNs in the absence of hematologic abnormalities. The patient’s presentation with SVT, splenomegaly, and a history of unprovoked pulmonary embolism aligns with reports of latent MPNs unmasked by thrombotic events, with JAK2 V617F detected in 32.7% of SVT cases and 49% of idiopathic SVT [6]. Molecular testing for JAK2 V617F was pivotal in establishing the diagnosis, highlighting its critical role in patients with unexplained thrombosis, particularly in younger individuals with recurrent events in unusual sites like the splanchnic veins [6]. This mutation has been postulated to drive the patient’s prothrombotic phenotype by promoting a pro-inflammatory state, cytokine production, and platelet/granulocyte activation [2-5].

Diagnosing occult MPNs in venous thromboembolism, particularly SVT, has historically relied on bone marrow biopsy, which has significant limitations [7]. Bone marrow biopsy, as performed in this case, is invasive, and its interpretation is semiquantitative, often failing to provide definitive evidence of MPN in latent cases [7]. Historically, endogenous erythroid colony (EEC) formation was included as a minor diagnostic criterion in international classifications until 2008 [8]. However, the identification of JAK2 mutations rendered EEC testing obsolete, as EEC formation results from the constitutive activation of erythropoietin receptor signaling driven by these mutations. Consequently, the 2016 World Health Organization classification removed EEC formation as a diagnostic marker for MPNs, replacing it with the more cost-effective, rapid, and standardized JAK2 V617F mutation testing [1]. In this case, bone marrow biopsy revealed normocellular marrow with moderate reticulin fibrosis but no overt MPN features; normal megakaryocyte morphology excluded pre-fibrotic MF, supporting a latent MPN diagnosis, with the 17% JAK2 V617F variant allele frequency likely explaining the absence of hematologic abnormalities [9].

The classification of JAK2 V617F-positive cases with normal blood counts is debated, potentially representing latent MPN or JAK2 V617F-associated clonal hematopoiesis of indeterminate potential (CHIP) [10]. The elevated risk of SVT in JAK2 V617F-associated CHIP may reflect its unique thrombotic predisposition driven by constitutive JAK/STAT signaling, as observed in MPNs [6]. Kristiansen et al. found that JAK2 V617F was 2.4 times more common in patients with ischemic stroke, often with CHIP and low mutation burden (<2%), indicating increased clotting risk without overt MPN [11]. 

Our management included thrombotic risk reduction and clonal process control. Indefinite anticoagulation with apixaban was critical due to the patient’s recurrent thrombosis, which was likely worsened by her semi-adherence to treatment. Low-dose aspirin (81 mg daily) was appropriately initiated to mitigate thrombosis recurrence, a standard approach in MPN-associated thrombophilia. While hydroxyurea is typically reserved for overt MPNs, its use in latent MPNs or JAK2 V617F-associated CHIP with high thrombotic risk is increasingly considered, though prospective data are lacking. The moderate reticulin fibrosis raises concerns for progression to MF, necessitating long-term monitoring for clonal evolution to overt MPN or acute myeloid leukemia.

The patient’s normal thrombophilia workup and absence of acquired risk factors reinforce JAK2 V617F as the primary thrombotic driver, with broader implications for hematologic cancers, and cardiovascular diseases [11-13]. The etiology of splenomegaly in our patient remains ambiguous, potentially driven by MPN or portal hypertension from chronic SVT. The absence of co-occurring mutations (e.g., DNMT3A, TET2, ASXL1) suggests JAK2 V617F as the sole driver, but future studies should explore such interactions. Prospective studies are needed to clarify the role of hydroxyurea and JAK inhibitors like ruxolitinib in latent MPNs or JAK2 V617F-associated CHIP and to define the natural history of these conditions.

## Conclusions

This case illustrates the critical role of JAK2 V617F molecular testing in diagnosing latent MPN in a young woman with recurrent SVT and normal blood counts. Her history of unprovoked thrombosis and splenomegaly, despite a bone marrow biopsy without overt MPN features, underscores the need to consider occult MPNs in similar patients. Tailored therapies, including anticoagulation, aspirin, and hydroxyurea, address thrombotic and clonal risks, with long-term follow-up essential for monitoring disease progression.

## References

[1] Khoury JD, Solary E, Abla O (2022). The 5th edition of the World Health Organization classification of haematolymphoid tumours: Myeloid and histiocytic/dendritic neoplasms. Leukemia.

[2] Levine RL, Wadleigh M, Cools J (2005). Activating mutation in the tyrosine kinase JAK2 in polycythemia vera, essential thrombocythemia, and myeloid metaplasia with myelofibrosis. Cancer Cell.

[3] Baxter EJ, Scott LM, Campbell PJ (2005). Acquired mutation of the tyrosine kinase JAK2 in human myeloproliferative disorders. Lancet.

[4] James C, Ugo V, Le Couédic JP (2005). A unique clonal JAK2 mutation leading to constitutive signalling causes polycythaemia vera. Nature.

[5] Kralovics R, Passamonti F, Buser AS (2005). A gain-of-function mutation of JAK2 in myeloproliferative disorders. N Engl J Med.

[6] Dentali F, Squizzato A, Brivio L (2009). JAK2V617F mutation for the early diagnosis of Ph- myeloproliferative neoplasms in patients with venous thromboembolism: a meta-analysis. Blood.

[7] Kiladjian JJ, Cassinat B (2023). Myeloproliferative neoplasms and splanchnic vein thrombosis: contemporary diagnostic and therapeutic strategies. Am J Hematol.

[8] Tefferi A, Vardiman JW (2008). Classification and diagnosis of myeloproliferative neoplasms: the 2008 World Health Organization criteria and point-of-care diagnostic algorithms. Leukemia.

[9] Moliterno AR, Kaizer H, Reeves BN (2023). JAK2 V617F allele burden in polycythemia vera: burden of proof. Blood.

[10] Steensma DP, Bejar R, Jaiswal S, Lindsley RC, Sekeres MA, Hasserjian RP, Ebert BL (2015). Clonal hematopoiesis of indeterminate potential and its distinction from myelodysplastic syndromes. Blood.

[11] Kristiansen MH, Kjær L, Skov V (2023). JAK2V617F mutation is highly prevalent in patients with ischemic stroke: a case-control study. Blood Adv.

[12] Jaiswal S, Fontanillas P, Flannick J (2014). Age-related clonal hematopoiesis associated with adverse outcomes. N Engl J Med.

[13] Liu W, Pircher J, Schuermans A (2024). Jak2 V617F clonal hematopoiesis promotes arterial thrombosis via platelet activation and cross talk. Blood.

